# Supplementary material for: Specific Composition Diets and Improvement of Symptoms of Immune-Mediated Inflammatory Diseases in Adulthood—Could the Comparison Between Diets Be Improved?
Source: Nutrients. 2025 Jan 29;17(3):493. doi: 10.3390/nu17030493 (PMC11819864; doi:10.3390/nu17030493)
Supplement: Supplementary file 1 [file nutrients-17-00493-s001.zip › nutrients-3422222-supplementary.docx]

| ***Table S1***  ***EMBASE search strategy*** | *Number of studies* |
| --- | --- |
| *autoimmune disease AND diet AND ([cochrane review]/lim OR [systematic review]/lim OR [meta analysis]/lim ) AND [1966-2024]/py* | *267* |
| *immune AND mediated AND disease AND diet AND ([cochrane review]/lim OR [systematic review]/lim OR [meta analysis]/lim) AND [1966-2024]/py* | *94* |
| *rheumatic AND disease AND diet AND ([cochrane review]/lim OR [systematic review]/lim OR [meta analysis]/lim) AND [1966-2024]/py* | *88* |
| *rheumatoid AND arthritis AND diet AND ([cochrane review]/lim OR [systematic review]/lim OR [meta analysis]/lim) AND [1966-2024]/py* | *237* |
| *spondyloarthritis AND diet AND ([cochrane review]/lim OR [systematic review]/lim OR [meta analysis]/lim ) AND [1966-2024]/py* | *18* |
| *psoriatic arthritis AND diet AND ([cochrane review]/lim OR [systematic review]/lim OR [meta analysis]/lim ) AND [1966-2024]/py* | *53* |
| *multiple and sclerosis AND diet AND ([cochrane review]/lim OR [systematic review]/lim OR [meta analysis]/lim ) AND [1966-2024]/py* | *122* |
| *Inflammatory and bowel and disease AND diet AND ([cochrane review]/lim OR [systematic review]/lim OR [meta analysis]/lim ) AND [1966-2024]/py* | *369* |
| *crohn´s AND disease AND diet AND [meta analysis]/lim AND [1966-2024]/py* | *115* |
| *crohn´s AND disease AND diet AND ([cochrane review]/lim OR [systematic review]/lim OR [meta analysis]/lim ) AND [1966-2024]/py* | *270* |
| *Ulcerative colitis AND diet AND ([cochrane review]/lim OR [systematic review]/lim OR [meta analysis]/lim ) AND [1966-2024]/py* | *217* |
| *Psoriasis AND diet AND ([cochrane review]/lim OR [systematic review]/lim OR [meta analysis]/lim ) AND [1966-2024]/py* | *95* |
|  | *1945* |
| ***MEDLINE search strategy.Meta-analysis/Systematic review (1992-2024 (09/12))*** |  |
| *((autoimmune disease) AND (diet)) AND (meta-analysis) AND (Systematic review))* | *78 88* |
| *((autoimmune disease) AND (diet)) AND (meta-analysis))* | *140* |
| *((rheumatic disease) AND (diet)) AND (meta-analysis) AND (Systematic review))* | *31* |
| *((rheumatic disease) AND (diet)) AND (meta-analysis))* | *59* |
| *((rheumatoid arthritis) AND (diet)) AND (meta-analysis) AND (Systematic review))* | *17* |
| *((rheumatoid arthritis) AND (diet)) AND (meta-analysis))* | *29* |
| *((spondyloarthritis) AND (diet)) AND (meta-analysis) AND (Systematic review))* | *4* |
| *((spondyloarthritis) AND (diet)) AND (meta-analysis))* | *4* |
| *((psoriatic arthritis) AND (diet)) AND (meta-analysis) AND (Systematic review))* | *4* |
| *((psoriatic arthritis) AND (diet)) AND (meta-analysis))* | *4* |
| *((multiple sclerosis) AND (diet)) AND (meta-analysis))* | *25* |
| *((multiple sclerosis) AND (diet)) AND (meta-analysis) AND (Systematic review))* | *18* |
| *((Inflammatory bowel disease) AND (diet)) AND (meta-analysis))* | *94* |
| *((Inflammatory bowel disease) AND (diet)) AND (meta-analysis) AND (Systematic review))* | *50* |
| *((crohn) AND (diet)) AND (meta-analysis))* | *76* |
| *((crohn) AND (diet)) AND (meta-analysis) AND (Systematic review))* | *41* |
| *((ulcerative colitis) AND (diet)) AND (meta-analysis))* | *44* |
| *((ulcerative colitis) AND (diet)) AND (meta-analysis) AND (Systematic review))* | *27* |
| *((Psoriasis) AND (diet)) AND (meta-analysis))* | *7* |
| *((Psoriasis) AND (diet)) AND (meta-analysis) AND (Systematic review))* | *6* |
|  | *768* |

**Table S2.** General characteristics of the Randomized Controlled Trials in Cochrane SR on the effect of diet on IMIDs

| **Cochrane SR** | **First author. Date** | **Duration. Months** | **Participants. Disease** | **Intervention/control.** | **Outcome** |
| --- | --- | --- | --- | --- | --- |
| Gwinnut et al 2022 | Skoldstam 2003(Cramp, 2013) | 3 | 51 RA | Cretan Mediterranean diet (MD) of de Lorgeril, et al for patients with RA/ An ordinary Western diet | Fatigue, pain, HAQ, DAS28, CRP, ESR |
|  | Sarzi-Puttini 2000 | 6 | 39 RA | Hypoallergenic diet (rice, cornmeal, cornbread, hydrolyzed milk, fresh pineapple, cooked apple) with no: wheal meal, eggs, milk, strawberries and acid fruit, tomato, chocolate, crustacean, dried fruit/ Same calorie content but containing allergenic food | Pain, ESR |
|  | Hafström 2001 | 12 | 66 RA | A vegan diet free of gluten/ Nonvegan diet – well-balanced | ACR 20 |
|  | Nenonen 1998 | 3 | 39 RA | Uncooked, lactobacilli rich, vegan diet/ Normal diet | DAS |
|  | Van de Laar 1992 | 3 | 94 RA | Allergy-additive-preservative-free diet/ Allergy free other than milk allergens and azo-colouring | Fatigue, CRP, ESR |
|  | Panush 1983 | 2,5 | 26 RA | A diet consisting of little meat, except fish and occasional fowl, no fruit, no herbs, no spices, no dairy products, no alcohol, no additives, no preservatives, supplemental iron, and vitamins/ Placebo diet – excluded select items from food groups, but included those eliminated from the experimental diet | Patient global VAS, TJC |
|  | Sundqvist 1982 | 2,5 | 10 RA | Fasted for 10 days, then Vegetarian diet with no alcohol, tobacco, or coffee/tea / Normal diet | TJC |
|  | Sköldstam1979 | 3 | 26 RA | Fasting for 7-10 days followed by Lacto-vegetarian diet/ No diet intervention | Pain, TJC |
| Limketkai et al., 2019   \|  \| Refined carbohydrate‐rich diet and unrestricted sugar intake \| \| --- \| --- \| | Brotherton 2014 | 1 | 7 active CD | High-fiber, low refined carbohydrates diet/ Low‐fiber diet | Induction of remission |
|  | Lomer 2001 | 4 | 20 active CD | Low microparticle diet/ Control: foods containing dietary microparticles were not discouraged. |  |
|  | Lomer 2005 | 4 | 83 active CD | Low calcium Low microparticles/Low calcium Normal microparticles/ Normal calcium Low microparticles/ Normal calcium Normal microparticles. |  |
|  | Bartel 2008 | 6 | 18 active CD | Highly restricted, organic diet/ Low-fiber, low-fat, and high-carbohydrate diet |  |
|  | Dariel 2007 |  | 51 active CD | Symptoms-guided diet/ Conventional nutritional advice |  |
|  | Lomer 2005 | 4 | 83 active CD | Low calcium Low microparticles/ Low calcium Normal microparticles/ Normal calcium Low microparticles/ Normal calcium Normal microparticles. |  |
|  | Bartel 2008 | 6 | 14 active CD | Highly restricted, organic diet/ Low-fiber, low-fat, and high-carbohydrate diet | Surrogate inflammatory biomarker – CRP, ESR |
|  | Dariel 2007 |  | 51 active CD | Symptoms-guided diet/ Conventional nutrition advice | Health-related quality of life - IBDQ. |
|  | Bartel 2008 | 6 | 14 active CD | Highly restricted, organic diet/ Low-fiber, low-fat, and high-carbohydrate diet |  |
|  | Brandes 1981 |  | 11 Inactive CD. | Low refined carbohydrate diet with increased intake of protein and fat/ High carbohydrate diet with reduced intake of protein and fat | Clinical relapse |
|  | Lorenz-Meyer 1996 |  | 204 Inactive CD | Low‐carbohydrate diet of less than 84 g/day/ Omega‐3 fatty acid capsules and general nutrition guidelines/ Placebo and general nutrition guidelines |  |
|  | Ritchie 1987 | 24 | 352 Inactive CD | Unrefined, fiber‐rich diet/ Refined carbohydrate‐rich diet and unrestricted sugar intake |  |
|  | Jones 1985 | 6 | 20 Inactive CD | Symptoms-guided diet/ Unrefined carbohydrate fiber‐rich diet |  |
|  | Riordan 1993 | 24 | 78 Inactive CD | Symptoms-guided diet/ General dietary advice and prednisolone taper |  |
|  | Albenberg 2018 | 12 | 214 Inactive CD | Low red, processed meat diet/ Moderate red and processed meats |  |
|  | Mutlu 2016 | 12 | 54 Inactive CD | Anti‐IBD diet (exclusion diets- low disaccharides, grains, saturated fats, red and processed meats-) and placebo supplement / Fructooligosaccharide supplement and "placebo diet"/"Placebo diet" and placebo supplement |  |
|  | Riordan 1993 | 24 | 78 Inactive CD | Symptoms-guided diet/ General dietary advice and prednisolone taper | CRP, ESR |
|  | Jones 1985 |  | 17 Inactive CD | Symptoms-guided diet/ Unrefined carbohydrate fiber‐rich diet | ESR |
|  | Candy 1995 | 1,5 | 21 active UC | Symptoms-guided diet/ Usual diet | Induction of remission |
|  | Keshteli 2016 | 6 | 28 inactive UC | Anti-inflammatory diet (Alberta-based)/ Diet based on Canada's Food Guide | Clinical relapse. |
|  | Bhattacharyya 2017 | 12 | 28 inactive UC | Carrageenan‐free diet + placebo/ Carrageenan free diet+ Carragean-containing capsules |  |
|  | Strisciuglio 2013 | 12 | 49 inactive UC | Milk-free diet/ Usual diet |  |
|  | Bhattacharyya 2017 | 12 | 15 inactive UC | Carrageenan‐free diet + placebo/ Carrageenan free diet+ Carragean-containing capsules | FC, TNF-α., IL6, IL8, SIBDQ |
| Parks et al 2020 | Irish 2017 | 3 | 17 RRMS | Modified Paleolithic diet. Gluten-Free/control: typical physician recommendation for MS | Physical and mental MSQoL |
|  | [Rezapour](https://www.cochranelibrary.com/cdsr/doi/10.1002/14651858.CD004192.pub4/references#CD004192-bbs2-0021)‐Firouzi 2013 | 6 | 65 RRMS | Co-supplemented hemp seed and evening primrose oils and advised Hot-nature diet / Supplement olive oil/ Co-supplemented hemp seed and evening primrose oils. | EDSS |
|  | [Yadav 2016](https://www.cochranelibrary.com/cdsr/doi/10.1002/14651858.CD004192.pub4/references#CD004192-bbs2-0029) | 12 | 61 RRMS | Very low-fat plant-based Diet / Control group: typical physician recommendation for MS | MFIS |

IMID: Immune-mediated inflammatory disease. VAS: Visual Analogue scale. HAQ: disease activity score. DAS28: Change in disease activity score 28. DAS: Disease activity score. CRP: C-Reactive Protein. ESR: erythrocyte sedimentation rate. ACR 20: American College of Rheumatology 20. TJC: Tender joint count. FC: Fecal calprotectin, TNF-α: Tumor necrosis factor-α. IL: Interleukin. SIBDQ: short IBD questionnaire. IBDQ: Inflammatory Bowel Disease Questionnaire. MSQoL: Multiple Sclerosis Quality of Life.

**Table S3.** General characteristics of the Clinical trials in the meta-analyses on the effect of diet on IMIDs

| **Meta-analysis: first author** | **RCT:**  **first author, date, study** | **Duration. Months** | **Participants. Disease** | **Intervention/control.** | **Outcome** |
| --- | --- | --- | --- | --- | --- |
| *Schönenberger SR-MA 2021 (62)* | Adam, 2003  RCT | 3 | 60, RA | AID (modified lactovegetarian diet)/ control: WD (usual diet in industrialized countries) | Pain, CRP |
|  | García Morales, 2020  RCT | 6 | 71, RA | MED (individualized according to Harris-Benedict BMR)/ control: general nutritional recommendations | Pain, CRP, ESR, DAS28, HAQ |
|  | Hafstrom, 2001  RCT | 12 | 66, RA | Gluten-free vegan diet/control: non vegan diet | Pain, CRP, HAQ |
|  | Kjeldsen-Kragh, 1991  RCT | 13 | 53, RA | Vegetarian/ control: ordinary mixed food | Pain, HAQ |
|  | Nenonen,1998  RCT | 2-3 | 42, RA | Vegan/ control: previous omnivorous diet | Pain, CRP, ESR |
|  | Skoldstam, 1979  RCT | 2 | 26, RA | Vegetarian/ control: normal diet | Pain, ESR |
|  | Skoldstam, 2003  RCT | 3 | 51, RA | Cretan MED/control: ordinary hospital food, and usual diet at home | Pain, CRP, ESR, HAQ |
| *Genel SR-MA 2020 (63)* | Skoldstam, 2003  RCT | 3 | 51, RA | Cretan MED/control: ordinary hospital food, and usual diet at home | Pain, CRP, ESR, HAQ |
| Turk SR-MA 2023 (64) | Holst-Hensen 1998  RCT | 1 | 30, RA | A liquid artificial peptide diet /control: usual food | Pain, HAQ |
|  | Winkvist 2018  RCT | 2,5 | 50, RA | A portfolio diet based on several food items with suggested anti-inflammatory effects/ control diet | Pain, DAS28 |
|  | Sarzi-Puttini 2000 RCT | 6 | 50, RA | An experimental diet high in unsaturated fats, low in saturated fats with hypoallergenic foods/ control: a well-balanced diet. | Pain |
|  | Skoldstam 2003  RCT | 3 | 51, RA | Cretan MED/control: ordinary hospital food, and usual diet at home. | Pain, HAQ, DAS 28 |
|  | Elkan 2008  RCT | 3 | 66, RA | A vegan diet free of gluten/control: a well-balanced non-vegan diet. | HAQ, DAS28 |
| Comeche SR-MA 2020 (66) | Lomer 2001  RCT | 4 | 20, ACT CD | Low Microparticles Diet /control: Normal Microparticles Diet | CDAI |
|  | Lomer 2005 RCT | 4 | 83, ACT CD | Diets: Low calcium Low microparticles, Low calcium Normal microparticles, Normal calcium Low microparticles, Normal calcium Normal microparticles. | CDAI |
|  | Chiba 2010  UNRCT | 1,5 | 22, ACT CD | Semi-vegetarian diet/ control: an omnivorous diet | CDAI, CRP, Albumin |
|  | Rajendran 2010 UNRCT | 1 | 29, REM CD | Immunoglobulin exclusion diet | CDAI |
|  | Marlow 2013 UNRCT | 1,5 | 8, ACT CD | Mediterranean-inspired anti-inflammatory diet | CRP |
|  | Konijeti 2017  UNRCT | 2,75 | 15, Act CD y UC | Autoimmune protocol diet (elimination of grains, legumes, nightshades, dairy, eggs, coffee, alcohol, nuts and seeds, refined/ processed sugars, oils, and food additives) | CRP, Albumin |
|  | Pedersen 2017  RCT | 1,5 | 89, REM IBD | Low FODMAP diet/ Normal diet | FC, CRP |
|  | Jian 2018  RCT | 6 | 97, REM UC | Immunoglobulin exclusion diet/Normal diet | Alb |
|  | Halmos 2016  RCT | 0,75 | 9, REM CD | TAD, Low FODMAP diet/typical Australian diet/  Normal diet | FC |
|  | Komperod 2017 NRCT | 0,5 | 12, REM CD | Komperod elimination diet/ control: Normal diet | FC |
| Zhan 2018 | Halmos 2016  RCT | 0,75 | 9, REM CD | TAD, Low FODMAP diet/typical Australian diet/  Normal diet | Diarrhea |
|  | Pedersen 2017  RCT | 1,5 | 89, REM IBD | Low FODMAP diet/ Normal diet | Diarrhea, AP, AB |
|  | Croagh 2007  UNRCT | 1,5 | 12, IBD | Low FODMAP diet/ Normal diet | Diarrhea |
|  | Prince 2016  UNRCT | 10 | 88, IBD | Low FODMAP diet/ Normal diet | Diarrhea, AP, AB,  Nausea, fatigue, constipation |
|  | Maagaard 2016 UNRCT | 1,5-2 | 49, IBD | Low FODMAP diet/ Normal diet | Diarrhea, AP,  AB,  Nausea, fatigue, constipation |
|  | Gearry 2008 UNRCT | 3-6 | 72, IBD Persistent abdominal symptoms | Low FODMAP diet | Diarrhea, AP  AB,  Nausea, fatigue, constipation |
| Peng 2022 | Pedersen 2017  RCT | 1,5 | 89, REM IBD | Low FODMAP diet/ Normal diet | Overall GI symptoms in IBD patients |
|  | Halmos 2016  RCT | 0,75 | 9, REM CD | TAD, Low FODMAP diet/typical Australian diet/  Normal diet | Overall GI symptoms in IBD patients |
|  | Bodini 2019  RCT | 1,5 | 51 REM IBD or mild disease activity | Low FODMAP diet/ Normal diet | Overall GI symptoms in IBD patients |
|  | Cox 2020 RCT | 1 | 52 IBD ongoing gut symptoms | Low FODMAP diet/ Placebo sham diet | Overall GI symptoms in IBD patients |
|  | Croagh 2007  UNRCT | 1,5 | 12, IBD | Low FODMAP diet | Overall GI symptoms in IBD patients |
|  | Prince 2016  UNRCT | 10 | 88, IBD | Low FODMAP diet | Overall GI symptoms in IBD patients |
|  | Maagaard 2016 UNRCT | 1,5-2 | 49, IBD | Low FODMAP diet | Overall GI symptoms in IBD patients |
|  | Gearry, 2008  UNRCT | 3-6 | 72, IBD Persistent abdominal symptoms | Low FODMAP diet | Overall GI symptoms in IBD patients, GI symptoms in CD and CU patients |
|  | Joyce 2014 UNRCT | 1,5 | 35, Inactive IBD and functional bowel disorders | Low FODMAP diet | Overall GI symptoms in IBD patients |
| Guerrero Aznar 2022 | Mousavi-Shirazi-Fard 2020 RCT | 3 | 100 RRMS | Modified anti-inflammatory diet/control: (WHO) healthy diet | EDSS, MFIS |
|  | Katz Sand 2019 RCT | 6 | 36 MS (28RRMS, 3SPMS,1PPMS) | Modified Mediterranean dietary program /control: Western-style diet | EDSS |
|  | Choi 2016  RCT | 6 | 48 RRMS | Fasting-Mimicking diet for 7 days, followed by a Mediterranean diet for 6 months/ Low Glycemic Load Ketogenic Diet (KD) / control: Criteria of a regular diet in German-population | EDSS, Physical MSQoL, Mental MSQoL |
|  | Irish 2017  RCT | 3 | 17 RRMS | Modified-Paleolithic diet. Gluten-Free / control: typical physician recommendation for MS | Physical MSQoL, Mental MSQoL |
|  | Rezapour‐Firouzi 2013 RCT | 6 | 65RRMS | Co-supplemented hemp seed and evening primrose oils and advised a Hot-nature diet. B: Supplement olive oil C: Co-supplemented hemp seed and evening primrose oils R | EDSS |
|  | Yadav 2016 RCT | 12 | 61 RRMS | Very low-fat plant-based Diet / Control group: typical physician recommendation for MS | MFIS |
|  | Bohlouli 2021 RCT | 6 | 147 RRMS | Modified Mediterranean diet:/ Traditional Iranian diet | EDSS, MFIS |
|  | Platero 2020  RCT | 4 | 51 MS | Mediterranean type food-pattern, supplemented /control group: The same isocaloric diet and placebo | EDSS |
| Snetselaar 2023 | Mousavi-Shirazi-Fard 2020 RCT | 3 | 100 RRMS | Modified anti-inflammatory diet (Anti-inflammatory) / control: (WHO) healthy diet | MFIS, Physical MSQoL, Mental MSQoL |
|  | Katz Sand 2019 RCT | 6 | 36 MS (28RRMS, 3SPMS,1PPMS) | Modified Mediterranean dietary program (Mediterranean) /control: Western-style diet | NFI-MS, Physical MSQoL, Mental MSQoL |
|  | Choi 2016  RCT | 6 | 48 RRMS | Fasting-Mimicking diet for 7 days, followed by a Mediterranean diet for 6 months (Fasting)/ Ketogenic/ Usual diet | MFIS, Physical MSQoL, Mental MSQoL |
|  | Irish 2017  RCT | 3 | 17 RRMS | Modified-Paleolithic diet. Gluten-Free (paleolithic) / control: typical physician recommendation for MS | FSS, Physical MSQoL, Mental MSQoL |
|  | Yadav 2016 RCT | 12 | 61 RRMS | Very low-fat plant-based Diet (Low fat) / Control group: typical physician recommendation for MS | MFIS, SF36-physical SF36-mental |
|  | Bohlouli 2021 RCT | 6 | 147 RRMS | Modified Mediterranean diet (Mediterranean):/ Traditional Iranian diet | MFIS, Physical MSQoL, Mental MSQoL |
|  | Fitzgerald 2018  RCT | 2 | 36 RRMS | Daily calorie restriction (CR) vs. Intermittent calorie restriction (Fasting) vs. Control diet | FAMS |
|  | Lee 2020  RCT | 3 | Mixed: RRMS: 1 PRMS: 1 SPMS: 10 PPMS: 2 | Modified Paleolithic diet (Paleolithic)/ Ketogenic/ Control diet | MFIS, Physical MSQoL, Mental MSQoL |
|  | Razeghi-Jahromi 2020 RCT | 12 | 80 RRMS | Mediterranean-like diet (Mediterranean)/ control diet: Standard healthy diet | MFIS |
|  | Roman 2020 RCT | 6 | 24 RRMS | Time-restricted feeding (Fasting)/ Usual care | Promis Fatigue |
|  | Wahls 2021  NRCT | 6 | 87 RRMS | Modified-Paleolithic elimination diet (Paleolithic)/Low-saturated fat diet (Low-fat) | MFIS, Physical MSQoL, Mental MSQoL |
|  | Yadav 2016 RCT | 12 | 61 RRMS | Very low-fat plant-based Diet / Control group: typical physician recommendation for MS | MFIS, SF36-physical, SF36-mental |

IMID: Immune-mediated inflammatory disease. RCT: Randomized Controlled Trial. NRCT: Randomized No Controlled Trial. UNRCT: No Randomized No Controlled Trial. AID Anti-inflammatory diet. MED: Mediterranean. HAQ: disease activity score. DAS28: change in disease activity score 28. CRP: C-Reactive Protein. ESR: erythrocyte sedimentation rate. FC: fecal calprotectin. MFIS: Modified Fatigue Impact Scale. MSQoL: Multiple Sclerosis Quality of Life. EDSS: Expanded Disability Status Scale. AP: abdominal pain. AB abdominal bloating. GI: gastrointestinal. IBD: inflammatory bowel disease. FODMAP: fermentable oligo-, di-, monosaccharides, and polyols. FAMS: functional assessment of multiple sclerosis- fatigue. TAP: typical Australian diet
